# MiR-146a participates in regulating the progression of periodontitis through the Wnt/β-catenin signaling pathway

**DOI:** 10.1371/journal.pone.0330739

**Published:** 2025-08-28

**Authors:** Shuixian Gao, Caiqin Mu, Juan Feng, Xiang Sun

**Affiliations:** Department of stomatology, The First Hospital of Yulin, Yulin, Shaanxi, China; Universidade Federal Fluminense, BRAZIL

## Abstract

**Objectives:**

This study aims to investigate the potential role of miR-146a/Wnt/β-catenin signaling axis in the pathogenesis of periodontitis, using LPS-stimulated hPDLCs as a cell model.

**Methods:**

Saliva samples were collected from the subjects and qRT-PCR was used to detect the expression of miR-146a and β-catenin in saliva. Clinical parameters, including probing depth (PD) and attachment loss (AL), were measured and their correlation with miR- 146a and β-catenin levels was determined. Cell proliferation capacity was assessed through CCK-8 assay and the production of inflammatory cytokines was evaluated through ELISA kits. Cell cycle distribution was detected by flow cytometry, and gene expression was detected by qRT-PCR and Western blot.

**Results:**

Our research indicates that compared with the control group, the CP group shows a higher miR-146a expression and a lower β-catenin expression in saliva (*P* < 0.0001). The expression of miR-146a is positively correlated with AL and PD (*P* < 0.001), and the expression of β-catenin is negatively correlated with AL and PD (*P* < 0.001). Inhibiting the expression of miR-146a can promote cell proliferation by regulating cell cycle distribution, reduce the production of inflammatory cytokines, inhibit the expression of p21 and promote the expression of CDK2 and CyclinD1. However, overexpression of miR-146a can result in the opposite effect. In LPS-stimulated hPDLCs, miR-146a expression is up-regulated while β-catenin expression is down-regulated. In addition, overexpression of miR-146a can inhibit β-catenin expression in cells. Simultaneously inhibiting the expression of miR-146a and β-catenin can reverse the effect of inhibiting miR-146a alone on alleviating LPS-induced cell damage.

**Conclusion:**

miR-146a can inhibit LPS-induced damage to hPDLCs by regulating the Wnt/β-catenin signaling pathway.

## Introduction

Periodontitis is a common chronic inflammatory non communicable disease and a major cause of tooth loss worldwide. Clinically, periodontitis is characterized by gingival inflammation, periodontal pocket formation, alveolar bone absorption, and gradual loosening of teeth. Pathologically, periodontitis involves multiple factors, mainly caused by microorganisms in dental plaque [1,2]. Specifically, G anaerobic bacteria and surface toxin lipopolysaccharides (LPS) have toxic effects on target cells and can lead to inflammatory immune responses [3]. The current therapeutic goal for periodontitis is to eliminate local pathogenic factors and control clinical inflammation. Common treatment methods include periodontal surgery, non-surgical treatments (such as tooth cleaning and root canal planning), and adjuvant therapy [4,5]. In addition, ample evidence has shown that periodontitis is closely related to systemic diseases, including cancer, hypertension, cardiovascular disease, and dementia [6–9]. Therefore, it is of great clinical significance to elucidate the potential pathogenesis of periodontitis, and improve its symptoms and related diseases.

miRNAs are a class of endogenous, highly conserved single stranded non coding small RNAs, approximately 22 nucleotides in size. miRNAs can down-regulate gene expression at the post transcriptional level by complementing the 3 ‘non transcriptional region (3’UTR) of mRNA, leading to mRNA degradation or translation inhibition. With the continuous development of RNA omics and bioinformatics prediction, over 530 types of RNA have been discovered in the human body, which have important implications for human diseases. The miR-146 family consists of miR-146a and miR-146b, which are located on different chromosomes and differ in mature forms by two nucleotides [10]. Despite the structural similarities, miR-146a and miR-146b have distinct biological activities. Previous studies have shown that under LPS stimulation, the mature form of miR-146a is produced in a nuclear factor kappa B dependent manner, leading to negative regulation of innate immune responses by down-regulating pro-inflammatory cytokines, chemokines, and other inflammatory mediators [11,12]. miR-146a is a key regulatory factor for LPS tolerance [13], which refers to the low response state of monocytes to subsequent LPS attacks after long-term exposure to LPS, thereby controlling the intensity and duration of inflammation. Motedayyen et al. found that compared with healthy individuals, patients with chronic periodontitis showed a higher miR-146a expression in periodontal tissues, and miR-146a expression was positively correlated with their clinical parameters [14]. Tang et al. found that miR-146a plays a regulatory role in the secretion of pro-inflammatory cytokines by LPS-induced human periodontal ligament fibroblasts through the TRAF6/p38 MAPK pathway. Maintaining the homeostasis of miR-146a plays a key role in controlling the inflammatory response of periodontal tissues [15]. Therefore, we believe that the down-regulation or impaired function of miR-146a may be associated with invasive periodontitis.

Wnts proteins, a class of secreted glycoproteins conserved in various organisms, can regulate the growth and development of many organs and tissues [16,17]. The Wnt signaling pathway can be divided into the canonical Wnt pathway that determines cell fate and the non canonical Wnt pathway that controls cell movement and tissue polarity [18,19]. The classic Wnt/β – catenin signaling pathway is activated by the binding of Wnt ligands to their specific receptors Frizzled (FZD) and low-density lipoprotein receptor associated protein 5/6 (LDL Receptor Related Protein 5/6, LRP5/6). Research has found that Wnt/β-catenin signaling pathway is mainly related to oxidative stress and inflammatory pathways. Up-regulation of the Wnt/β-catenin signaling pathway can reduce inflammatory response in rheumatoid arthritis and diabetes periodontitis [20,21]. Down-regulation of the Wnt/β-catenin signaling pathway can exacerbate the migration, invasion, and inflammatory response of fibroid synovial cells in rheumatoid arthritis [22]. Liu et al. showed that down-regulation of miR-146a inhibited jawbone osteoporosis in ovariectomized rats by regulating the Wnt/β – Catenin signaling pathway [23]. However, the interaction between miR-146a and the Wnt/β-catenin pathway in periodontitis remains unclear.

Therefore, this study aims to explore the role of miR-146a/Wnt/β-catenin signaling axis in periodontitis, and how it affects the progression of periodontitis by regulating inflammation and proliferation, thereby providing new therapeutic strategies for periodontitis.

## Materials and methods

### Sample collection

From January 2023 and January 2024, 35 patients with chronic periodontitis admitted to our hospital were selected as the CP group, and 35 healthy volunteers undergoing physical examinations at our hospital were selected as the control group. All participants signed informed consent forms prior to the study, and the experimental protocol was approved by the Ethics Committee of Yulin First Hospital (Ethics Number: 20230106070). There was no significant difference in age and gender between the two groups (*P* > 0.05). The probing depth (PD) and attachment loss (AL) of periodontium were recorded in detail. Inclusion criteria: ① No history of smoking, drinking, or taking special medications; ② Meeting the diagnostic criteria for chronic periodontitis; ③ Mucosal diseases that do not involve the gums, such as oral leukoplakia, oral lichen planus, etc; ④ Cooperating with relevant inspections to obtain complete data. Exclusion criteria: ① Accompanied by systemic diseases; ② Administration of antibiotics and immunosuppressants within 3 months before admission; ③ Pregnant or lactating women.

Saliva samples were collected from the two groups in the morning. The specific method is as follows: the two groups were fasted from solids and liquids for 4 hours before collection, and their mouthes were wiped with a cotton swab dipped in 2% citric acid. About 1 mL of saliva was collected using a sterile RNAase-free centrifuge tube and centrifuged at 3000 r/min for 15 minutes at 4 °C. The saliva supernatant was taken out for later use.

### Cell isolation and culture

Human periodontal ligament cells (hPDLC) were purchased from the American Type Culture Collection (Manassas, VA, USA) and inoculated into Dulbecco Modified Eagle Medium (DMEM) containing 10% fetal bovine serum, 100U/ml penicillin and 100 μg/ml streptomycin, then stored with 5%CO_2_ at 37°C. LPS was obtained from InvivoGen, and cells were treated with 1 μg/ml or 10 μg/ml LPS for 6, 12, and 24 hours, respectively [24].

### Cell transfection

Sub-confluent cell monolayers (70%−80% density) in 6-well plates were transfected using cationic lipid-based non-viral vectors (Lipofectamine 3000, Carlsbad, CA, USA). Plasmid constructs included pcDNA-miR-146a, shRNA-miR-146a, miR-146a mimics, pcDNA-β-catenin, sh-β-catenin (Shanghai Genepharma), each co-delivered with their respective negative controls (Shanghai Genepharma). Complex formation involved pre-incubation of 2.5 μg nucleic acids with 5 μL transfection reagent in serum-free DMEM for 20 min at ambient temperature. Post-addition to cultured cells, the medium was replaced with complete growth supplements after 6 hours. Transfection efficiency was quantitatively assessed at 48 hours through concurrent GFP co-expression via flow cytometry (BD FACSVerse).

### Cell counting kit-8 (CCK-8) assay

hPDLCs were synchronized in serum-free medium for 24 hours before seeding in 96-well microplates (5 × 10³ cells/well) with complete α-MEM containing 10% FBS. After 12-hour attachment, proliferative capacity was evaluated at 0, 24, 48, and 72-hour intervals. At each timepoint, 10 μL of water-soluble tetrazolium salt WST-8 reagent (Cell Counting Kit-8, Dojindo, Japan) was introduced followed by 2-hour incubation at 37°C in a humidified 5% CO₂ atmosphere. The absorbance was quantified by measuring light absorption characteristics at dual wavelengths (450 nm primary detection, 620 nm reference) using a multimode plate reader (SpectraMax i3x).

### Enzyme-linked immunosorbent assay (ELISA)

Freshly isolated cell suspensions underwent ice-cold lysis utilizing for 30 minutes. Following centrifugation at 12,500 × g (4°C, 20 min), supernatants underwent protein quantification via the bicinchoninic acid approach (Thermo Fisher Scientific). Cytokine (TNF – α, IL-1 β, IL-6, and IL-8) measurements employed ELISA kits (Solarbio, China), with standardized aliquots (25 μg total protein per well) loaded onto pre-coated 96-well plates in quadruplicate. After sequential incubations with detection antibodies (1 hr, 25°C) and streptavidin-polyHRP conjugates (45 min), chromogenic development involved 3,3’,5,5’-tetramethylbenzidine substrate (15 min dark incubation). The enzymatic reaction was halted with 1 N H₂SO₄, and optical densities were determined at dual wavelengths (450 nm primary, 630 nm reference) using a SpectraMax i3x platform.

### Cell cycle

The cell cycle distribution was measured by flow cytometry. Cells were seeded in 6-well culture plates at optimal density (1 × 10⁵ cells/well) and transfected for exactly 48 hours.Post-trypsinization using 0.25% EDTA-trypsin, cells underwent triple PBS washes before fixation in pre-chilled 75% ethanol. Subsequent permeabilization employed 0.25% Triton X-100/PBS for 15 min, followed by dual-staining with RNase-treated propidium iodide and Annexin V-FITC in dark conditions. The cell cycle distribution was analyzed by flow cytometry using CellQuest software (Becton Dickinson).

### Real-time quantitative PCR (RT-qPCR)

Total RNA was extracted using TRIzol™ Reagent (Thermo Fisher Scientific). RNA integrity/purity (A₂₆₀/A₂₈₀ = 1.8–2.0) was confirmed electrophoretically. cDNA was synthesized from 1 μg RNA using SuperScript™ III (Invitrogen) with Oligo(dT)₂₀ primers. SYBR Green qPCR (Biosharp) reactions (10 μL) contained: 2 μL cDNA template; 0.2 μM gene-specific primers (forward/reverse); 5 μL 2 × master mix; Nuclease-free water to volume. Cycling: 95°C/3 min; 40 cycles of 95°C/30 s → 60°C/30 s → 72°C/30 s. Reactions included triplicates and no-template controls. Relative expression levels were measured using the 2^-ΔΔCt^ method. Primers used in our study are listed in Table 1.

**Table 1:** Primer sequences.

Genes	Primers(5’-3’)
β-catenin	F: AAAATGGCAGTGCGTTTAG
	R: TTTGAAGGCAGTCTGTCGTA
miR-146a	F:ACACTCCAGCTGGGTGAGAACTGAATTCCA
	R:CTCAACTGGTGTCGTGGAGTCGGCAATTCAGTTGAGAACCCATG
GAPDH	F:CCTCAAGATTGTCAGCAAT
	R:CCATCCACAGTCTTCTGAGT

### Western blot

Protein samples were obtained using RIPA lysis buffer (Beyotime, China) and protein concentrations were analyzed by BCA (Sigma, USA). The same amount of protein was separated by 15%SDS-PAGE and transferred on the PVDF membrane.

Membranes were incubated with primary antibodies against CDK2 (1:1000, Thermo Fisher Scientific), CyclinD1 (1:1000, Thermo Fisher Scientific), P21 (1:10000, Abcam), and GAPDH (1:10000, Abcam). After rinsing with TBST for three times, the membrane was incubated with an appropriate second antibody (1:3000, Cell signaling technology) overnight at 4°C. Protein signals were measured using the ECL chemiluminescence kit (EMD Millipore). Image J was used to quantify protein bands.

### Statistical analysis

All experiments were repeated at least three times independently. Data was presented as mean ± standard deviation (Mean ± SD). Student’s t-test or Mann-Whitney test was used to compare normally distributed and non-normally distributed groups, respectively. One-way ANOVA was used to analyze the differences between multiple groups, followed by Bonferroni’s multiple comparison tests or t-test as needed. Pearson test (in the case of normal distribution) or Spearman test (in the case of non-normal distribution) was used to determine the correlation coefficients of data. All statistical analyses were conducted on GraphPad 8.0 software. *P* < 0.05 was considered statistically significant.

## Results

### Expression of miR-146a and β-catenin in saliva of subjects and their relationship with disease severity

**Fig 1:**
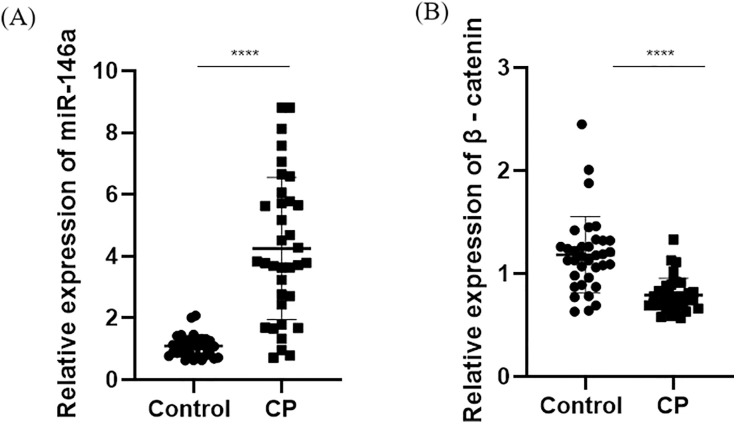
Relative expression levels of miR-146a and β-catenin in saliva of subjects. **(A)** The expression of miR-146a was determined by qRT-PCR. ****p < 0.0001 versus control. **(B)** The expression of β-catenin was determined by qRT-PCR. ****p < 0.0001 versus control. qRT-PCR: Quantitative Real-time Reverse Transcriptase Polymerase Chain Reaction.

**Fig 2:**
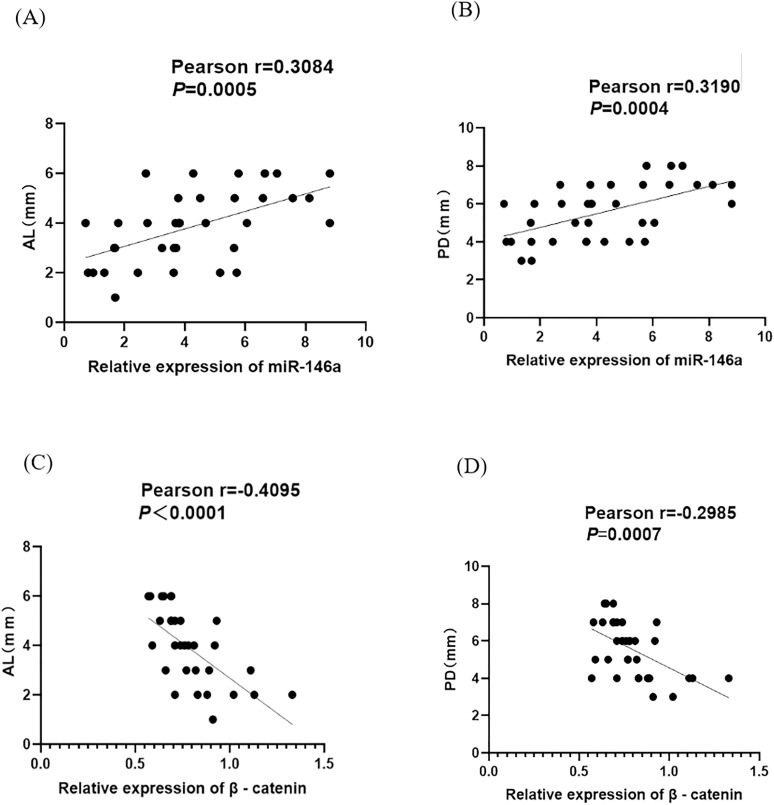
The relationship between the expression levels of miR-146a andβ -catenin and AL and PD in CP patients. **(A)** Pearson regression analysis between miR-146a expression level and AL. **(B)** Pearson regression analysis between miR-146a expression level and PD. **(C)** Pearson regression analysis between β-catenin expression level and AL. **(D)** Pearson regression analysis between β-catenin expression level and PD. CP: Chronic periodontitis. AL: Attachment loss. PD: Probing depth.

Compared with the control group, the expression of miR-149a in saliva is significantly increased and the expression of β-catenin is significantly decreased in the CP group (Fig 1A–1B). A correlation test was performed to determine the association between miR-146a and β-catenin levels and disease severity. Pearson regression analysis shows a linear positive correlation between miR-146a and AL and PD, while a linear negative correlation between β-Catenin and AL and PD, with statistical significance (*P* < 0.05) (Fig 2A–2D).

### MiR-146a level is increased in LPS-stimulated hPDLCs

**Fig 3:**
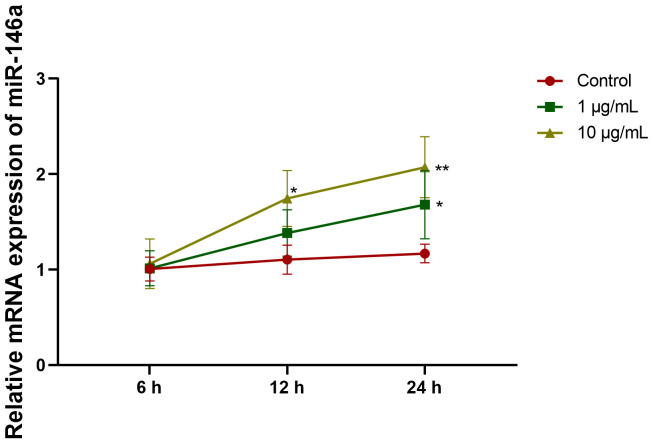
miR-146a levels increase in LPS-stimulated hPDLCs. hPDLCs was treated with LPS (1 and 10 μg/ml) for 6, 12, and 24 hours. Then, the expression level of miR-146a in each group was measured using qRT-PCR. *p < 0.05 versus control, #*p* < 0.05 versus NC inhibitor. LPS: Lipopolysaccharide. hPDLCs: Human periodontal ligament cells. qRT-PCR: Quantitative Real-time Reverse Transcriptase Polymerase Chain Reaction.

In LPS-stimulated hPDLC, miR-146a level is elevated. hPDLC was treated with LPS (1 and 10 μg/ml) for 6, 12, and 24 hours, respectively. Compared with the control group, miR-146a level increases in hPDLCs stimulated with 1 μg/ml or 10 μg/ml LPS for 12 hours and 24 hours. Therefore, in the following experiment, 1 μg/ml LPS was used to stimulate hPDLCs for 24 hours (Fig 3).

### Effect of miR-146a knockout on the proliferation and inflammation of hPDLCs

**Fig 4:**
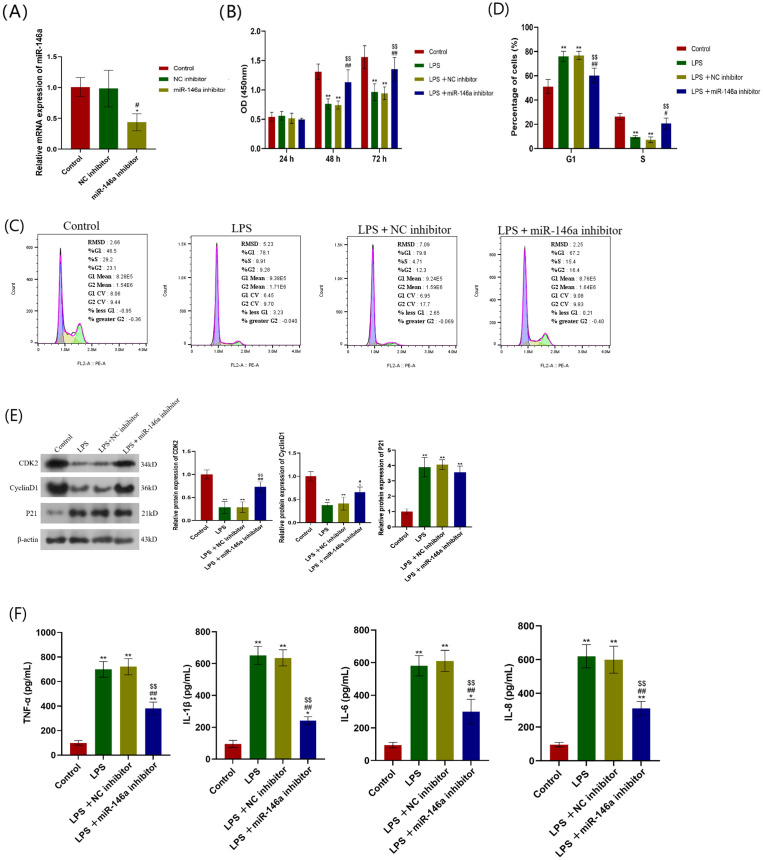
Effect of miR-146a inhibitor on hPDLCs proliferation and inflammation. (A) hPDLCs was transfected with miR-146a inhibitor and its negative control, and the transfection efficiency was determined by qRT-PCR. *P < 0.05 versus control; #*P* < 0.05 versus NC inhibitor. (B) hPDLCs was stimulated with LPS and transfected with miR-146a inhibitor and its negative control. The cell proliferation capacity of hPDLCs at 24, 48 and 72 h was measured using CCK-8. **(C)** Cell cycle determination by flow cytometry analysis. **(D)** Quantification of cell cycle distribution. **(E)** The protein expression levels of CDK2, p21 and cyclin D1 were determined by Western blot. **(F)** ELISA was used to measure concentrations of inflammatory cytokines TNF-α, IL-1β, IL-6, and IL-8. *,**P < 0.05, 0.01versus control; #, ##*P* < 0.05, 0.01versus LPS; $, $$ *P* < 0.05, 0.01versus LPS + NC inhibitor. hPDLCs: Human periodontal ligament cells. qRT-PCR: Quantitative Real-time Reverse Transcriptase Polymerase Chain Reaction. CCK-8: Cell Counting Kit-8. ELISA: Enzyme-linked immunosorbent assay.

To determine the role of miR-146a under LPS stimulation, miR-146a inhibitor was transfected into hPDLCs. The results show that compared with the negative control (NC inhibitor), transfection with miR-1466 inhibitor can significantly reduce miR-16a expression (Fig 4A). CCK-8 assay shows that the cell proliferation capacity is significantly decreased after LPS stimulation, which can be significantly reversed by miR-146a inhibitor (Fig 4B). Flow cytometry was used to analyze the cell cycle. The results show that LPS stimulation can increase G1 phase cells while decrease S phase cells in hPDLCs. However, the LPS-induced effect can be partially reversed by miR-146a inhibitor (Fig 4C–4D). In addition, the protein expression of CDK2 and cyclin D1 decreases after LPS stimulation, while increases after using miR-146a inhibitor. The protein expression of p21 increases after LPS stimulation, while decreases after using miR-146a inhibitor (Fig 4E). Furthermore, ELISA was used to detect the levels of inflammatory cytokines, including TNF-α, IL-1β, IL-6, and IL-8, the concentration of which increases under LPS stimulation while significantly decreases after transfection with miR-146a inhibitor (Fig 4F).

### Effects of overexpression of miR-146a on proliferation and inflammation of hPDLC

**Fig 5:**
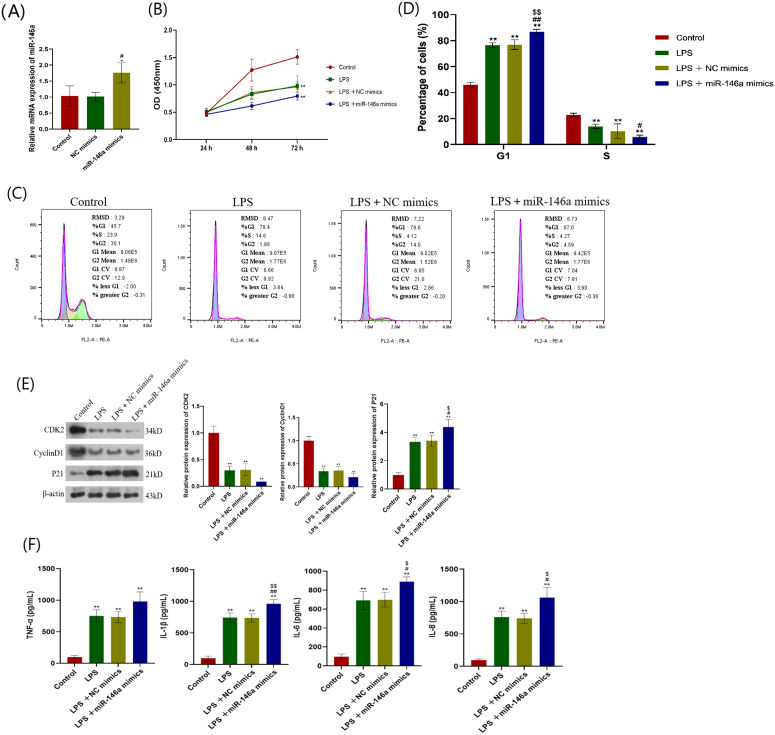
Effect of miR-146a mimics on hPDLCs proliferation and inflammation. (A) hPDLCs was transfected with miR-146a mimics and its negative control, and the transfection efficiency was determined by qRT-PCR. *P < 0.05versus control; #*P* < 0.05 versus NC mimics. (B) hPDLCs was stimulated with LPS and transfected with miR-146a mimics and its negative control. The cell proliferation capacity of hPDLCs at 24, 48 and 72 h was measured using CCK-8. **(C)** Cell cycle determination by flow cytometry analysis. **(D)** Quantification of cell cycle distribution. **(E)** The protein expression levels of CDK2, p21 and cyclin D1 were determined by Western blot. **(F)** ELISA was used to measure concentrations of inflammatory cytokines TNF-α, IL-1β, IL-6, and IL-8. *,**P < 0.05, 0.01versus control; #, ##*P* < 0.05, 0.01versus LPS; $, $$*P* < 0.05, 0.01versus LPS + NC mimics. hPDLCs: Human periodontal ligament cells. qRT-PCR: Quantitative Real-time Reverse Transcriptase Polymerase Chain Reaction. CCK-8: Cell Counting Kit-8. ELISA: Enzyme-linked immunosorbent assay.

In addition, transfection of miR-146a mimics into hPDLCs results in a significant decrease in miR-146a expression (Fig 5A). Contrary to previous results, it was found that miR-146a mimics can reduce cell proliferation capacity and increase the percentage of G1 phase cells (Fig 5B–5D). Besides, miR-146a mimics can reduce the protein expression of CDK2 and cyclin D1 and increase the protein expression of p21 (Fig 5E). Furthermore, the concentrations of TNF-α, IL-1β, IL-6, and IL-8 were detected after transfection with miR-146a mimics (Fig 5F).

### miR-146a regulates β-catenin-mediated transcriptional activity in hPDLCs

**Fig 6:**
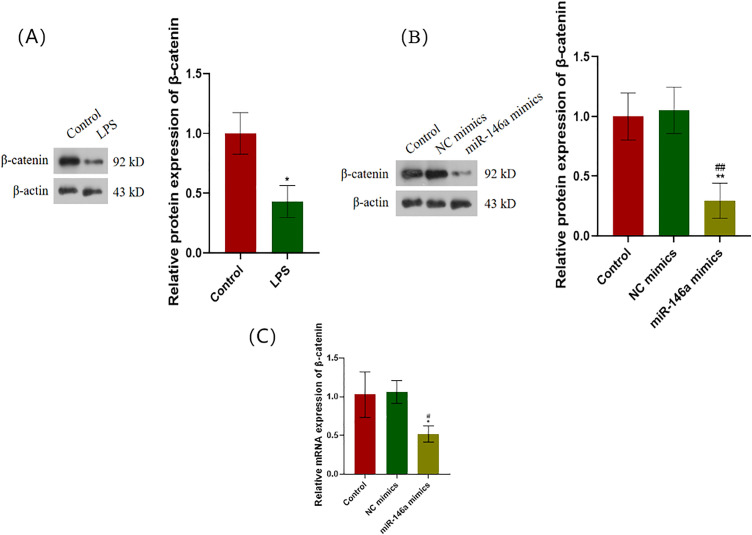
β-catenin is a functional mediator for miR-146a in hPDLCs. (A) Protein expression levels of β-catenin were measured using Western blot after hPDLCs were stimulated by LPS. *P < 0.05 versus control. (B) Protein expression of β-catenin was measured by Western blot when transfected with miR-146a mimics. (C) mRNA levels of β-catenin were measured using qRT-PCR when transfected with miR-146a mimics. *P < 0.05 versus control; #*P* < 0.05 versus NC mimics. hPDLCs: Human periodontal ligament cells. LPS: Lipopolysaccharide. qRT-PCR: Quantitative Real-time Reverse Transcriptase Polymerase Chain Reaction.

After LPS stimulation of hPDLCs, β-catenin expression decreases (Fig 6A). Compared with the control group, the mRNA and protein expression levels of β-catenin are down-regulated after transfection with miR-146a mimics (Fig 6B, 6C).

### miR-146a/Wnt/β-catenin regulates LPS-stimulated proliferation and inflammation of hPDLCs

**Fig 7:**
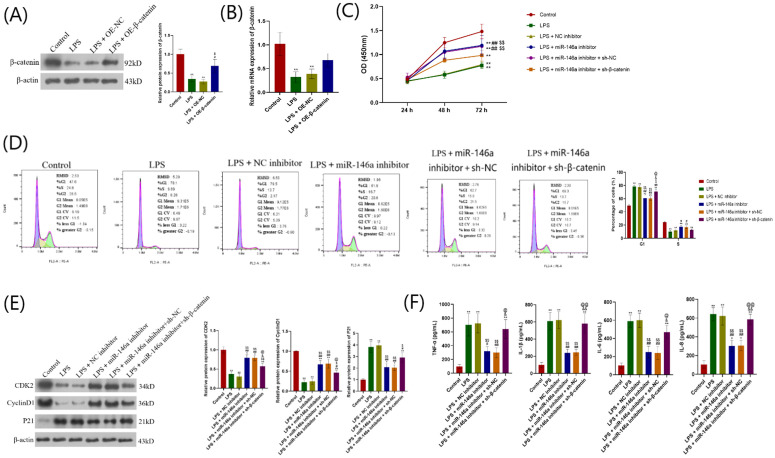
miR-146a/Wnt/β-catenin regulates LPS-stimulated hPDLCs proliferation and inflammation. (A) When β-catenin is overexpressed in LPS-induced hPDLCs, the protein expression level of β-catenin is measured. **P < 0.01versus control; #*P* < 0.05 versus LPS; $*P* < 0.05 versus LPS + OE-NC. (B) The mRNA level of β-catenin was measured when β-catenin was overexpressed in LPS-induced hPDLCs. **P < 0.01versus control. (C) hPDLCs were stimulated with LPS and co-transfected with miR-146a inhibitor, pcDNA-β-catenin, and/or their respective controls. Cell proliferation ability of hPDLCs at 24, 48, and 72 hr was determined using CCK-8 assay. (D) Cell cycle determination and quantification of cell cycle distribution by flow cytometry analysis. (E) The protein expression levels of CDK2, p21 and cyclin D1 were determined by Western blot. (G) ELISA was used to measure the expression of TNF-α, IL-1β, IL-6, and IL-8. *,**P < 0.05, 0.01versus control; #, ##*P* < 0.05, 0.01versus LPS; $, $$*P* < 0.05, 0.01versus LPS + NC inhibitor; &, &&*P* < 0.05, 0.01versus LPS + miR-146a inhibitor; @, @@P<0.05, 0.01versus LPS + miR-146a inhibitor+sh-NC. LPS: Lipopolysaccharide. hPDLCs: Human periodontal ligament cells. ELISA: Enzyme-linked immunosorbent assay.

Next, the effects of miR-146a and β-catenin on LPS-stimulated hPDLCs were analyzed. pcDNA β-catenin transfection leads to increased β-catenin expression in LPS-stimulated hPDLCs (Fig 7A, 7B). CCK-8 and flow cytometry show that inhibiting of miR-146a expression can significantly enhance cell proliferation by promoting cell progression from G1 phase to S phase, decrease p21 expression and increase cyclin D1 expression. Inflammation can be alleviated by reducing the production of TNF-α, IL-1β, IL-6, and IL-8 in LPS-stimulated hPDLCs. However, these results are significantly reversed after inhibiting β-catenin expression. Since inhibited β-catenin expression can reverse the improvement of cell proliferation induced by miR-146a inhibitor and increase the production of inflammatory cytokines (Fig 7C–7F).

## Discussion

Periodontitis is a non-specific infectious disease mainly caused by G-anaerobic bacteria such as Porphyromonas gingivalis and Actinobacillus actinomycetes. Periodontitis poses a serious threat to human health, with more than half of patients experiencing tooth loss. There is currently no effective method for preventing and treating periodontitis. Previous studies have shown that inflammation serves as the main factor exacerbating periodontitis, and the surrounding inflammatory microenvironment indirectly reduces the generative activity of gingival tissue [25,26]. Therefore, the key treatment for periodontitis lies in alleviating inflammation and restoring the damaged gingival cell function in gingivitis [27].

LPS has been reported to be one of the major causes of periodontitis, with a strong toxic effect on periodontal tissue cells. HPDLCs, the main cells of the periodontal membrane, play a key role in tooth support, collagen production and tissue regeneration. In addition, hPDLCs, as target cells of LPS, are commonly used for in vitro stimulation in periodontitis environment. In the present study, 1 μg/ml LPS was used to stimulate an inflammatory periodontal cell model.

The currently highly-studied miRNA is a type of endogenous non-coding small molecule RNA with high conservation, mainly functions by binding to the 3 ‘untranslated region of target genes. Research has confirmed a large number of differentially expressed miRNAs in the blood, saliva, and periodontal tissue cells of patients with periodontitis. Na et al. screened differentially expressed miRNAs in periodontal tissue cells through miRNA gene chip detection, and found that miR-128 was significantly overexpressed [28]. Further verification revealed that miR-128 can affect the expression of TNF-α through the p38/MAPK signaling pathway, thereby regulating the inflammatory response in vivo. Hong et al. transfected miR-200c into human embryonic palatal mesenchymal cells and detected osteogenic differentiation markers within the cells [29]. The results showed that the expression levels of osteocalcin, calcium, and other substances were significantly increased, directly affecting the absorption and regeneration of alveolar bone. In addition, miR-200c can significantly down-regulate relevant inflammatory factors such as IL-6 and IL-8. Together, differentially expressed miRNAs have significant regulatory effects in periodontal disease. Previous studies have found that mRNA-146a plays an important role in inflammation regulation, and changes in its levels are related to the pathogenesis of inflammatory diseases [30,31]. Xie et al. conducted research using gene chip technology and found that some miRNAs such as miRNA-146a were up-regulated in gingival tissues of patients with chronic periodontitis [32], indicating that miRNA-146a is closely related to the pathogenesis of chronic periodontitis. Mahendra et al. revealed that miR-146a was up-regulated in the subgingival plaque samples from patients with generalized aggressive periodontitis. Therefore, miR-146a can serve as a risk biomarker for such patients [33]. Motedayyen et al. demonstrated that the level of miR-146a in patients with chronic periodontitis was higher than that in healthy individuals, while the levels of inflammatory factors TNF-α and IL-6 were reduced [34]. From the above, the positive correlation between miR-146a levels and clinical parameters indicates the pathophysiological role of miR-146a in chronic periodontitis. The present study validates the results of previous studies to some extent. In this study, miR-146a is up-regulated in LPS-stimulated hPDLCs. miR-146a knockout improves cell proliferation by regulating the cell cycle, particularly by promoting progression from G1 to S phase. In addition, miR-146a knockout inhibits inflammatory responses by reducing the production of inflammatory cytokines including TNF-α, IL-1β, IL-6, and IL-8. Overexpression of miR-146a has opposite effects on cell proliferation and inflammation. The above results indicate that mRNA-146a has a potential pathophysiological role in periodontitis. However, to date, the specific mechanism by which miR-146a regulates chronic periodontitis has not yet been fully elucidated.

Recent years have witnessed increasing attention to the correlation between the classic Wnt pathway and periodontitis. β-catenin is a core regulatory factor downstream of the classical Wnt pathway, and its expression in the cytoplasm is an important link in activating the classical pathway. Previous studies have found that the Wnt/β-catenin signaling pathway is mainly related to oxidative stress and inflammatory responses. Up-regulating the Wnt/β – catenin signaling pathway can reduce inflammatory responses in rheumatoid arthritis and periodontitis [20,35]. Yu et al found that β-catenin expression in periodontal tissue of rats with periodontitis was significantly reduced compared with the control group [36]. Naruse et al. demonstrated in animal experiments that the Wnt/β-catenin signaling pathway regulated the development of periapical periodontitis [37]. Results of the above research are consistent with those of the present study. In addition, Liu et al. confirmed that miR-146a can inhibit mandibular osteoporosis in ovariectomized rats by down-regulating the Wnt/β-catenin signaling pathway [38]. However, there are currently no reports on the role of miR-146a/Wnt/β-catenin signaling axis in periodontitis. In this study, β-catenin knockout partially reverses the inhibitory effect of miR-146a on LPS-induced cell damage. Although the exact role and mechanism of β-catenin have not been fully elucidated, the results of this study suggest that β-catenin is involved in the development of periodontitis and miR-146a can prevent periodontitis by regulating β-catenin.

## Conclusion

Overall, the present study suggests that miR-146a may be involved in LPS-stimulated inhibition of hPDLCs proliferation and inflammation by regulating the Wnt/β-catenin signaling pathway. However, this study still has certain limitations. Although our in vitro findings have elucidated the mechanism role of miR-146a in regulating the Wnt/β-catenin signaling pathway, the lack of animal validation makes it impossible to draw conclusions about its therapeutic applicability. Future studies should employ conditional knockout mice to track cell-type-specific effects and evaluate the dynamic interaction between miR-146a and Wnt/β-catenin signaling pathway within the complex pathophysiological microenvironment of periodontitis.

## Supporting information

S1 Fig As full as possible length gels and blots for Fig 2. (DOC)

S2 Fig As full as possible length gels and blots for Fig 3. (DOCX)

S3 Fig As full as possible length gels and blots for Fig 4. (DOCX)

S4 Fig As full as possible length gels and blots for Fig 5. (DOCX)

## References

[1] Dannewitz B, Holtfreter B, Eickholz P. Periodontitis—therapy of a widespread disease. Bundesgesundheitsblatt-Gesundheitsforschung-Gesundheitsschutz. 2021;64:931–40. 34236451 10.1007/s00103-021-03373-2 PMC8264996

[2] Slots J. Periodontitis: facts, fallacies and the future. Periodontol 2000. 2017;75(1):7–23. doi: 10.1111/prd.12221 28758294

[3] Wei C, Tan CK, Xiaoping H, Junqiang J. Acanthoic acid inhibits LPS-induced inflammatory response in human gingival fibroblasts. Inflammation. 2015;38(2):896–901. doi: 10.1007/s10753-014-0051-7 25373915

[4] Fischer RG, Lira Junior R, Retamal-Valdes B, Figueiredo LC de, Malheiros Z, Stewart B, et al. Periodontal disease and its impact on general health in Latin America. Section V: Treatment of periodontitis. Braz Oral Res. 2020;34(supp1 1):e026. doi: 10.1590/1807-3107bor-2020.vol34.0026 32294679

[5] Sanz M, Herrera D, Kebschull M, Chapple I, Jepsen S, Beglundh T, et al. Treatment of stage I-III periodontitis-The EFP S3 level clinical practice guideline. J Clin Periodontol. 2020;47 Suppl 22(Suppl 22):4–60. doi: 10.1111/jcpe.13290 32383274 PMC7891343

[6] Sanz M, Marco Del Castillo A, Jepsen S, Gonzalez-Juanatey JR, D’Aiuto F, Bouchard P, et al. Periodontitis and cardiovascular diseases: Consensus report. J Clin Periodontol. 2020;47(3):268–88. doi: 10.1111/jcpe.13189 32011025 PMC7027895

[7] Harding A, Robinson S, Crean S, Singhrao SK. Can Better Management of Periodontal Disease Delay the Onset and Progression of Alzheimer’s Disease? J Alzheimers Dis. 2017;58(2):337–48. doi: 10.3233/JAD-170046 28453484

[8] Barton MK. Evidence accumulates indicating periodontal disease as a risk factor for colorectal cancer or lymphoma. Wiley Online Library. 2017; 67: 173–4. 10.3322/caac.21367 28272812

[9] Merchant AT, Virani SS. Evaluating Periodontal Treatment to Prevent Cardiovascular Disease: Challenges and Possible Solutions. Curr Atheroscler Rep. 2017;19(1):4. doi: 10.1007/s11883-017-0640-7 28120314

[10] Lu L-F, Boldin MP, Chaudhry A, Lin L-L, Taganov KD, Hanada T, et al. Function of miR-146a in controlling Treg cell-mediated regulation of Th1 responses. Cell. 2010;142(6):914–29. doi: 10.1016/j.cell.2010.08.012 20850013 PMC3049116

[11] Rusca N, Monticelli S. MiR-146a in Immunity and Disease. Molecular Biology International. 2011;2011(8):437301. 22091404 10.4061/2011/437301 PMC3200075

[12] Taganov KD, Boldin MP, Chang K-J, Baltimore D. NF-kappaB-dependent induction of microRNA miR-146, an inhibitor targeted to signaling proteins of innate immune responses. Proc Natl Acad Sci U S A. 2006;103(33):12481–6. doi: 10.1073/pnas.0605298103 16885212 PMC1567904

[13] Nahid MA, Pauley KM, Satoh M, Chan EKL. miR-146a is critical for endotoxin-induced tolerance: IMPLICATION IN INNATE IMMUNITY. J Biol Chem. 2009;284(50):34590–9. doi: 10.1074/jbc.M109.056317 19840932 PMC2787321

[14] Motedayyen H, Ghotloo S, Saffari M, Sattari M, Amid R. Evaluation of MicroRNA-146a and Its Targets in Gingival Tissues of Patients With Chronic Periodontitis. J Periodontol. 2015;86(12):1380–5. doi: 10.1902/jop.2015.150319 26313020

[15] Tang L, Li X, Bai Y, Wang P, Zhao Y. MicroRNA-146a negatively regulates the inflammatory response to Porphyromonas gingivalis in human periodontal ligament fibroblasts via TRAF6/p38 pathway. J Periodontol. 2019;90(4):391–9. doi: 10.1002/JPER.18-0190 30378773

[16] Sylvie J, Ellen C, Kris V. The role of Wnt in cell signaling and cell adhesion during early vertebrate development. Front Biosci (Landmark Ed). 2011;16(6):2352–66. doi: 10.2741/3858 21622181

[17] Cabello J, Neukomm LJ, Günesdogan U, Burkart K, Charette SJ, Lochnit G, et al. The Wnt pathway controls cell death engulfment, spindle orientation, and migration through CED-10/Rac. PLoS Biol. 2010;8(2):e1000297. doi: 10.1371/journal.pbio.1000297 20126385 PMC2814829

[18] Ling L, Nurcombe V, Cool SM. Wnt signaling controls the fate of mesenchymal stem cells. Gene. 2009;433(1–2):1–7. doi: 10.1016/j.gene.2008.12.008 19135507

[19] Tu X, Joeng KS, Nakayama KI, Nakayama K, Rajagopal J, Carroll TJ, et al. Noncanonical Wnt signaling through G protein-linked PKCdelta activation promotes bone formation. Dev Cell. 2007;12(1):113–27. doi: 10.1016/j.devcel.2006.11.003 17199045 PMC1861818

[20] Tan W, Qiu Y, Chen N, Gao J, Liang J, Liu Y, et al. The intervention of intestinal Wnt/β-catenin pathway alters inflammation and disease severity of CIA. Immunol Res. 2021;69(4):323–33. doi: 10.1007/s12026-021-09190-8 34037945

[21] Dong L, Xu P. Danzhi Jiangtang capsule alleviate hyperglycemia and periodontitis via Wnt/β-catenin signaling in diabetic rat. J Tradit Chin Med. 2021;41(4):608–16. doi: 10.19852/j.cnki.jtcm.2021.03.013 34392654

[22] Cai L, Mu Y-R, Liu M-M, Zhou M-Y, Meng B, Liu F-Y, et al. Penta-acetyl Geniposide Suppresses Migration, Invasion, and Inflammation of TNF-α-Stimulated Rheumatoid Arthritis Fibroblast-Like Synoviocytes Involving Wnt/β-Catenin Signaling Pathway. Inflammation. 2021;44(6):2232–45. doi: 10.1007/s10753-021-01495-y 34101073

[23] Liu H, Yue X, Zhang G. Downregulation of miR‑146a inhibits osteoporosis in the jaws of ovariectomized rats by regulating the Wnt/β‑catenin signaling pathway. Int J Mol Med. 2021;47(3):6. doi: 10.3892/ijmm.2020.4839 33655338 PMC7834969

[24] Huang N, Li C, Sun W, Wu J, Xiao F. Long non-coding RNA TUG1 participates in LPS-induced periodontitis by regulating miR-498/RORA pathway. Oral Dis. 2021;27(3):600–10. doi: 10.1111/odi.13590 32762066

[25] Avinash K, Malaippan S, Dooraiswamy JN. Methods of Isolation and Characterization of Stem Cells from Different Regions of Oral Cavity Using Markers: A Systematic Review. Int J Stem Cells. 2017;10(1):12–20. doi: 10.15283/ijsc17010 28531913 PMC5488772

[26] Wang F, Chen X, Han Y, Xi S, Wu G. circRNA CDR1as Regulated the Proliferation of Human Periodontal Ligament Stem Cells under a Lipopolysaccharide-Induced Inflammatory Condition. Mediators Inflamm. 2019;2019:1625381. doi: 10.1155/2019/1625381 31582895 PMC6754938

[27] Jiang C, Wang Q, Song M, Wang M, Zhao L, Huang Y. Coronarin D affects TNF-α induced proliferation and osteogenic differentiation of human periodontal ligament stem cells. Arch Oral Biol. 2019;108:104519. doi: 10.1016/j.archoralbio.2019.104519 31470142

[28] Na HS, Park MH, Song YR, Kim S, Kim H-J, Lee JY, et al. Elevated MicroRNA-128 in Periodontitis Mitigates Tumor Necrosis Factor-α Response via p38 Signaling Pathway in Macrophages. J Periodontol. 2016;87(9):e173–82. doi: 10.1902/jop.2016.160033 27240473

[29] Hong L, Sharp T, Khorsand B, Fischer C, Eliason S, Salem A, et al. MicroRNA-200c Represses IL-6, IL-8, and CCL-5 Expression and Enhances Osteogenic Differentiation. PLoS One. 2016;11(8):e0160915. doi: 10.1371/journal.pone.0160915 27529418 PMC4987006

[30] Lukiw WJ, Zhao Y, Cui JG. An NF-kappaB-sensitive micro RNA-146a-mediated inflammatory circuit in Alzheimer disease and in stressed human brain cells. J Biol Chem. 2008;283(46):31315–22. doi: 10.1074/jbc.M805371200 18801740 PMC2581572

[31] Huang Y, Liu Y, Li L, Su B, Yang L, Fan W, et al. Involvement of inflammation-related miR-155 and miR-146a in diabetic nephropathy: implications for glomerular endothelial injury. BMC Nephrol. 2014;15:142. doi: 10.1186/1471-2369-15-142 25182190 PMC4236663

[32] Xie Y, Shu R, Jiang S, Liu D, Zhang X. Comparison of microRNA profiles of human periodontal diseased and healthy gingival tissues. Int J Oral Sci. 2011;3(3):125–34. doi: 10.4248/IJOS11046 21789961 PMC3470093

[33] Mahendra J, Mahendra L, Fageeh HN, Fageeh HI, Ibraheem W, Abdulkarim HH, et al. miRNA-146a and miRNA-126 as Potential Biomarkers in Patients with Coronary Artery Disease and Generalized Periodontitis. Materials (Basel). 2021;14(16):4692. doi: 10.3390/ma14164692 34443215 PMC8398247

[34] Motedayyen H, Ghotloo S, Saffari M, Sattari M, Amid R. Evaluation of MicroRNA-146a and Its Targets in Gingival Tissues of Patients With Chronic Periodontitis. J Periodontol. 2015;86(12):1380–5. doi: 10.1902/jop.2015.150319 26313020

[35] Lei D, Pei XU. Danzhi Jiangtang capsule alleviate hyperglycemia and periodontitis via Wnt/β-catenin signaling in diabetic rats. Journal of Traditional Chinese Medicine. 2021;41(4):608–16. 34392654 10.19852/j.cnki.jtcm.2021.03.013

[36] Yu M, Liu S, Yu J. Influence of Fupi Yishen Decoction on periodontal tissue damage in rats with periodontitis by regulating Wnt/β-catenin signaling pathway. J Hubei Univ Chinese Med. 2023;25(2):15–9.

[37] Naruse H, Itoh S, Itoh Y, Kagioka T, Abe M, Hayashi M. The Wnt/β-catenin signaling pathway has a healing ability for periapical periodontitis. Sci Rep. 2021;11(1):19673. doi: 10.1038/s41598-021-99231-x 34608236 PMC8490427

[38] Liu XZ, Yue X, Zhang G. Downregulation of miR-146a inhibits osteoporosis in the jaws of ovariectomized rats by regulating the Wnt/beta-catenin signaling pathway. Int J Mol Med. 2021;47(3):6. doi: 10.3892/ijmm.2020.4839 33655338 PMC7834969

