# Supplementary figures and images for: MiR-146a participates in regulating the progression of periodontitis through the Wnt/β-catenin signaling pathway
Source: PLoS One. 2025 Aug 28;20(8):e0330739. doi: 10.1371/journal.pone.0330739 (PMC12393756; doi:10.1371/journal.pone.0330739)

**S1 Fig. As full as possible length gels and blots for Figure 2.**


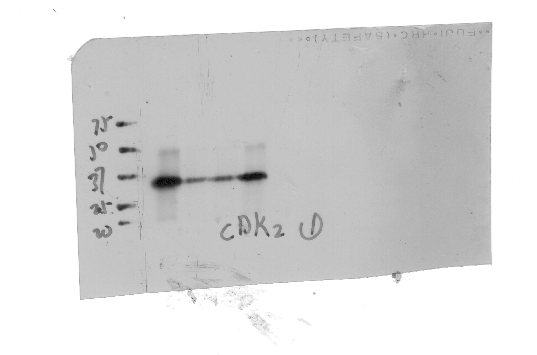


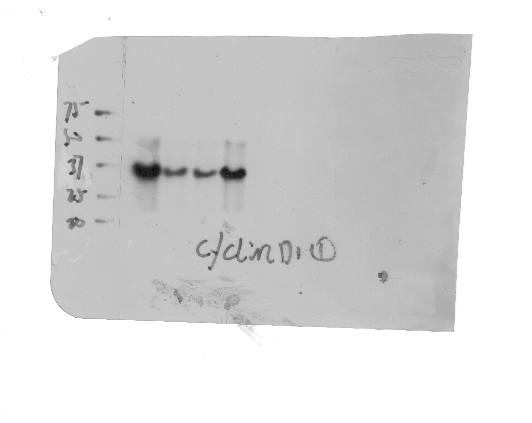


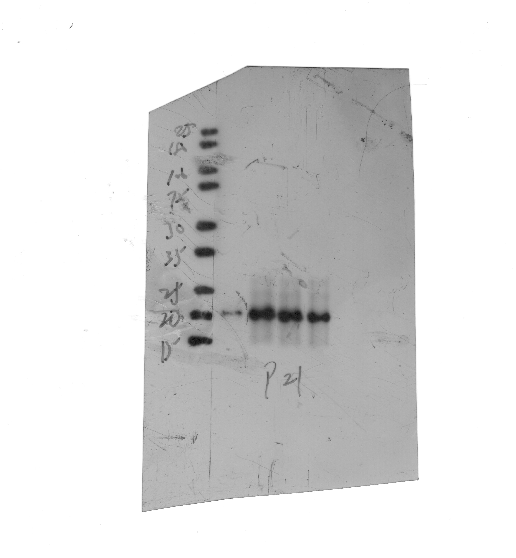


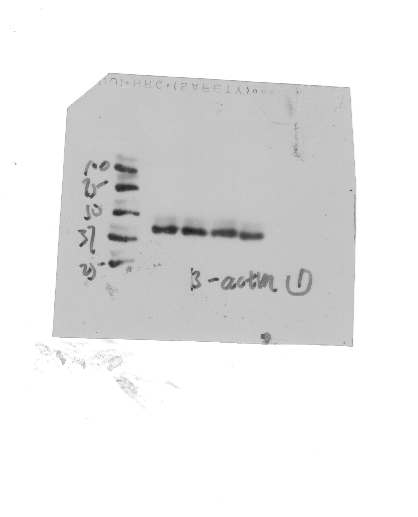

Supplement: S1 Fig — (DOC) [file pone.0330739.s001.doc]

**S2 Fig. As full as possible length gels and blots for Figure 3.**


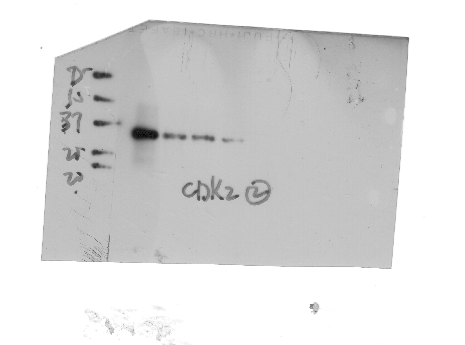


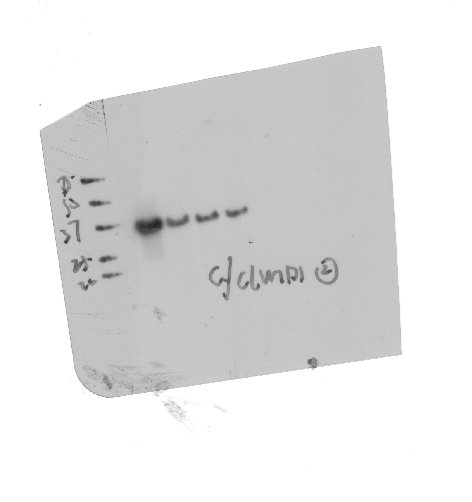


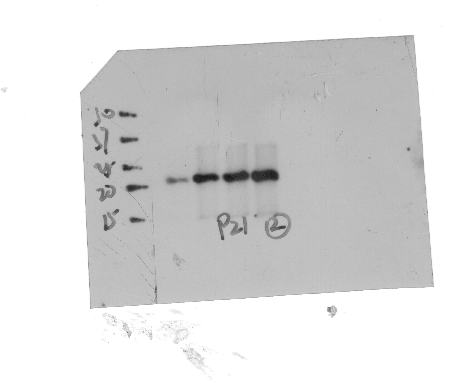


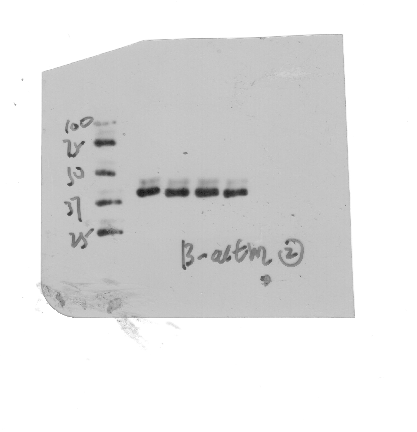

Supplement: S2 Fig — (DOCX) [file pone.0330739.s002.docx]

**S3 Fig. As full as possible length gels and blots for Figure 4.**


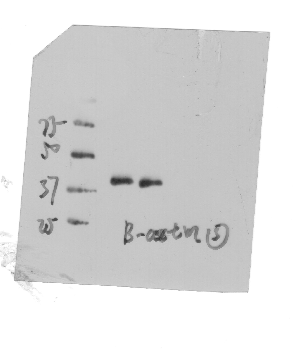


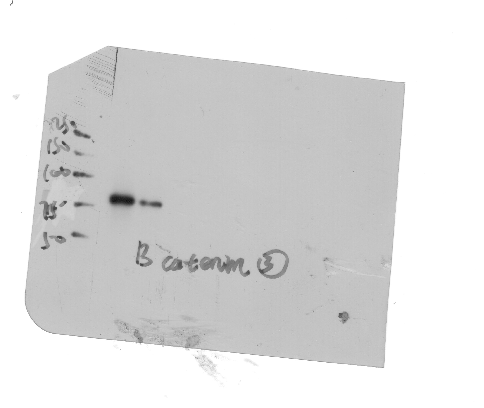


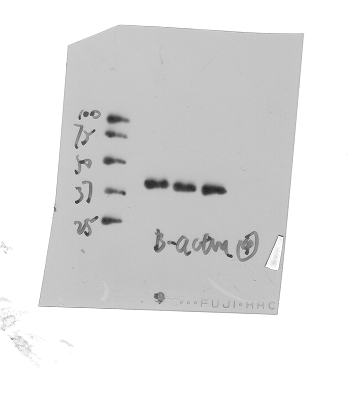


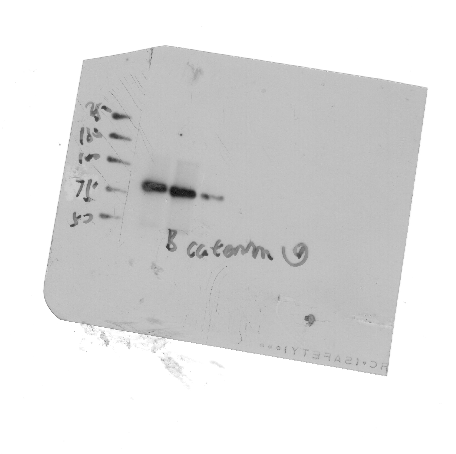

Supplement: S3 Fig — (DOCX) [file pone.0330739.s003.docx]

**S4 Fig. As full as possible length gels and blots for Figure 5.**


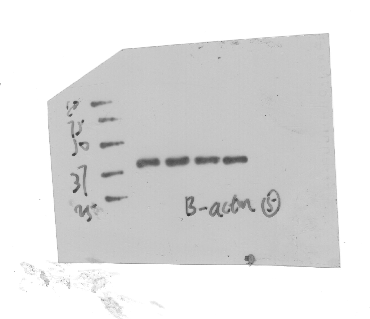


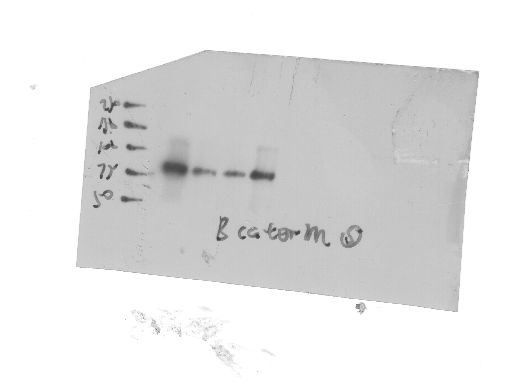


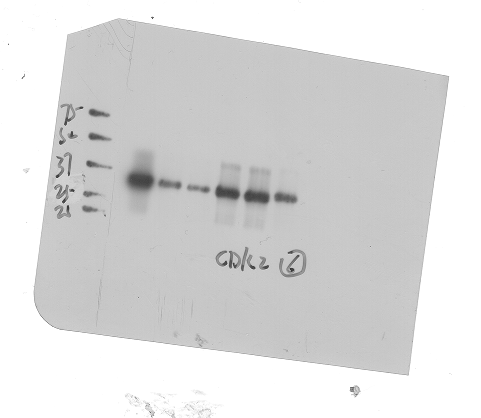


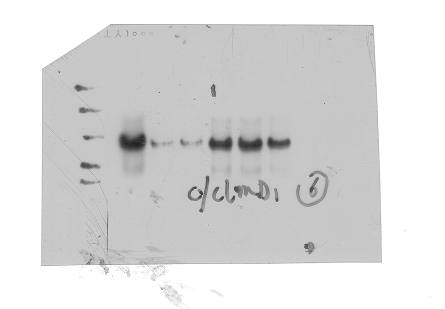


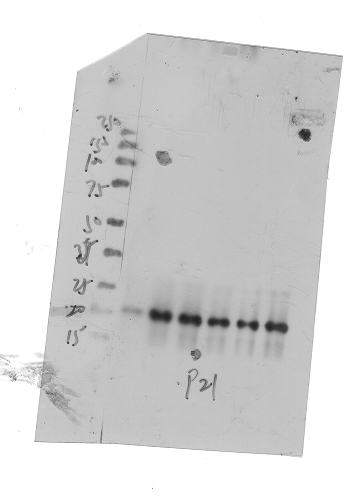


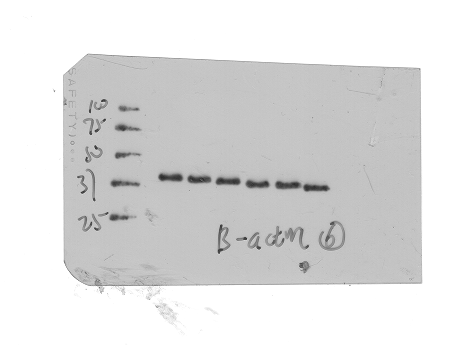

Supplement: S4 Fig — (DOCX) [file pone.0330739.s004.docx]
